# Tubular epithelial cells-derived small extracellular vesicle-VEGF-A promotes peritubular capillary repair in ischemic kidney injury

**DOI:** 10.1038/s41536-022-00268-x

**Published:** 2022-12-17

**Authors:** Xin Zhong, Tao-Tao Tang, An-Ran Shen, Jing-Yuan Cao, Jing Jing, Cui Wang, Xiao-Xiao Zhu, Yi Wen, Zuo-Lin Li, Bin Wang, Suo-Fu Qin, Bi-Cheng Liu, Lin-Li Lv

**Affiliations:** 1 Institute of Nephrology, Zhong Da Hospital, Southeast University School of Medicine, 87 Ding Jia Qiao Road, Nanjing, China; 2 Kexing Biopharm Co., Ltd, Floor 15-19, Building B, Chuangyi Technology Building, No. 198, Keji Middle 1st Road, Nanshan, Shenzhen, China

**Keywords:** Acute kidney injury, Translational research

## Abstract

Peritubular capillaries (PTCs) are closely related to renal tubules in structure and function, and both are pivotal regulators in the development and progression of acute kidney injury (AKI). However, the mechanisms that underlie the interaction between PTCs and tubules during AKI remain unclear. Here we explored a new mode of tubulovascular crosstalk mediated by small extracellular vesicles (sEV) after AKI. In response to renal ischemia/reperfusion (I/R) injury, endothelial proliferation of PTCs and tubular expression of vascular endothelial growth factor-A (VEGF-A) were increased, accompanied by a remarkable redistribution of cytoplasmic VEGF-A to the basolateral side of tubular cells. Meanwhile, the secretion mode of VEGF-A was converted in the injured tubular cells, which showed a much greater tendency to secrete VEGF-A via sEV other than the free form. Interestingly, tubular cell-derived VEGF-A-enriched sEV (sEV-VEGF-A) turned out to promote endothelial proliferation which was regulated by VEGF receptors 1 and 2. Furthermore, inhibition of renal sEV secretion by Rab27a knockdown resulted in a significant decrease in the proliferation of peritubular endothelial cells in vivo. Importantly, taking advantage of the newly recognized endogenous repair response of PTCs, exogenous supplementation of VEGF-A + sEV efficiently recused PTC rarefaction, improved renal perfusion, and halted the AKI to CKD transition. Taken together, our study uncovered a novel intrinsic repair response after AKI through renal tubule-PTC crosstalk via sEV-VEGF-A, which could be exploited as a promising therapeutic angiogenesis strategy in diseases with ischemia.

## Introduction

Acute kidney injury (AKI), a common clinical event with high morbidity and mortality, is associated with increased risks of chronic kidney disease (CKD) and end-stage renal disease (ESRD)^1,2^. Understanding the injury and repair processes of AKI is crucial for developing new therapeutic strategies to induce/enhance renal recovery. Regardless of etiology, proximal tubule injury as well as renal microvascular dysfunction, is the key pathological change of AKI^3^. Since the peritubular capillaries are situated in close contact with renal tubules, it is reasonable to presume that these two cell types communicate intimately, which may determine the prognosis of AKI^4^.

Proximal tubular epithelial cells (TECs) are highly susceptible to ischemic and toxic insults. After a mild or moderate injury, TECs usually have a perfect chance of recovery depending on the dedifferentiation and regeneration of surviving TECs^5,6^. However, persistent or severe injury may lead to incomplete tubular repair, which is detrimental to microvasculature^7–9^. On the other side, microvascular damage could cause permanent rarefaction of peritubular capillaries (PTCs), resulting in renal hypoxia and thereby aggravating the impaired recovery of TECs^10–13^. The vicious circle created by tubular injury and microvascular dysfunction promotes the transition of AKI to CKD^14,15^, which also suggests a pivotal cross-talk between the two neighboring cells. Hence, elucidating the underlying mechanism of TECs-PTCs communication will shed new light on manipulating the repair processes after AKI.

Small extracellular vesicles (sEV) are cell-derived membrane-enclosed vesicles of endosomal origin and are typically 30–150 nm in diameter^16^. Their roles in intercellular communication by transferring cargoes such as lipids, proteins, mRNAs, and microRNAs between cells have been well documented over the past decades. Our previous studies revealed that hypoxia and albumin can trigger TECs to release sEV-carrying microRNA-23a and CCL-2 mRNA, respectively, that have the tropism to macrophages and provoke tubulointerstitial inflammation^17,18^. Moreover, it was also reported that sEV from hypoxia-preconditioned TECs have the capacity to limit the activation of profibrotic genes and preventing loss of capillaries and tubulointerstitial fibrosis in post-ischemic kidneys^19,20^. Thus, TEC-derived sEV might function as an active vector, exhibiting essential roles in the pathogenesis of kidney disease. However, whether sEV contribute to the communication between tubules and PTCs during AKI remains unknown.

In this study, we have investigated sEV released from hypoxic TECs and their roles in the injury and repair processes of PTCs in a murine model of ischemic AKI. We found that hypoxia augmented the secretion of VEGF-A-enriched sEV (sEV-VEGF-A) from TECs, which was subsequently transferred to PTCs and promoted the proliferation and inflammatory response of endothelial cells in the ischemia-reperfusion (I/R) injured kidney. Moreover, adoptive treatment of sEV engineering with VEGF-A potently ameliorated the chronic progression of AKI via promoting PTCs repair. Our study revealed a novel mechanism of sEV-VEGF-A mediated crosstalk between tubules and PTCs, which represents an intrinsic repair response of angiogenesis after AKI.

## Results

### Tubular injury and PTC rarefaction in I/R-induced AKI

**Fig. 1:**
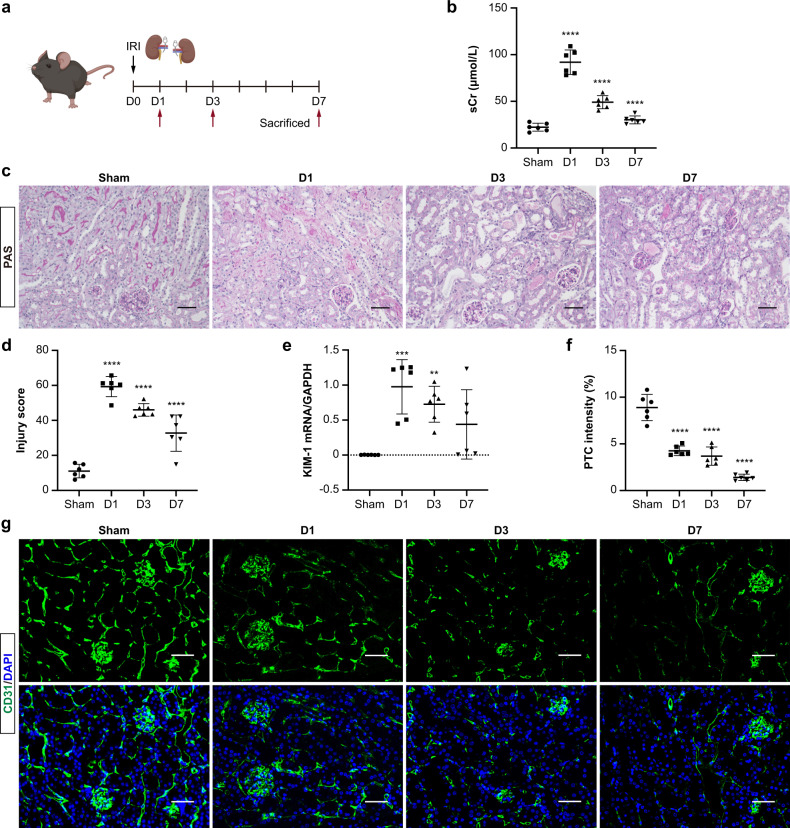
Tubular injury and PTCs rarefaction in I/R-induced AKI. **a** Schematic diagram of the experimental design. Mice were subjected to a 28-min bilateral renal ischemia-reperfusion (I/R) injury and were euthanized at 1, 3, and 7 days after I/R. Schematic created with BioRender.com. **b** Changes in serum creatinine (sCr) following I/R injury (*n* = 6). **c** Representative images of PAS-stained kidneys. Scale bars, 100 μm. **d** Quantification of kidney injury based on PAS staining (*n* = 6). **e** RT-qPCR analysis of KIM-1 mRNA levels in kidneys (*n* = 6). **f** and **g** Representative confocal images of CD31-stained kidney sections. Scale bars, 50 μm. Quantification of PTC intensity based on six mice, with at least ten sections counted in each. Data are presented as means ± SD. **p < 0.01, ***p < 0.001, ****p < 0.0001 vs. Sham group. One-way ANOVA.

An experimental model of AKI was induced by renal I/R injury to evaluate the damage and repair of renal tubules and PTCs (Fig. 1a). Serum creatinine (Fig. 1b) and tissue damage such as necrotic tubules (Fig. 1c and d) were strongly increased at day 1-post I/R and fell thereafter at days 3 and 7, indicating an endogenous tubular repair at the early stage of ischemic injury. Consistently, kidney injury molecule-1(Kim-1), a tubular damage marker, was significantly up-regulated in the I/R kidney at day 1, preceding a tendency of reduction at day 7 (Fig. 1e). Besides, the expression of inflammatory factors (TNF-α, MCP-1, VCAM-1, ICAM-1) was increased continually in the injured kidney (Supplementary Fig. 1). Next, the density of PTCs was evaluated by CD31-staining. Despite the rapid reparative response of tubules after I/R injury, capillary rarefaction started at day 1 and continued to deteriorate at days 3 and 7, suggesting failed recovery of PTCs (Fig. 1f and g). Hence, although renal function is recovered, kidney injury is sustained after AKI, especially PTCs damage is likely difficult to get repair, leading to PTC rarefaction.

### Endothelial cell proliferation of PTCs and tubular expression of VEGF-A are induced after I/R injury

**Fig. 2:**
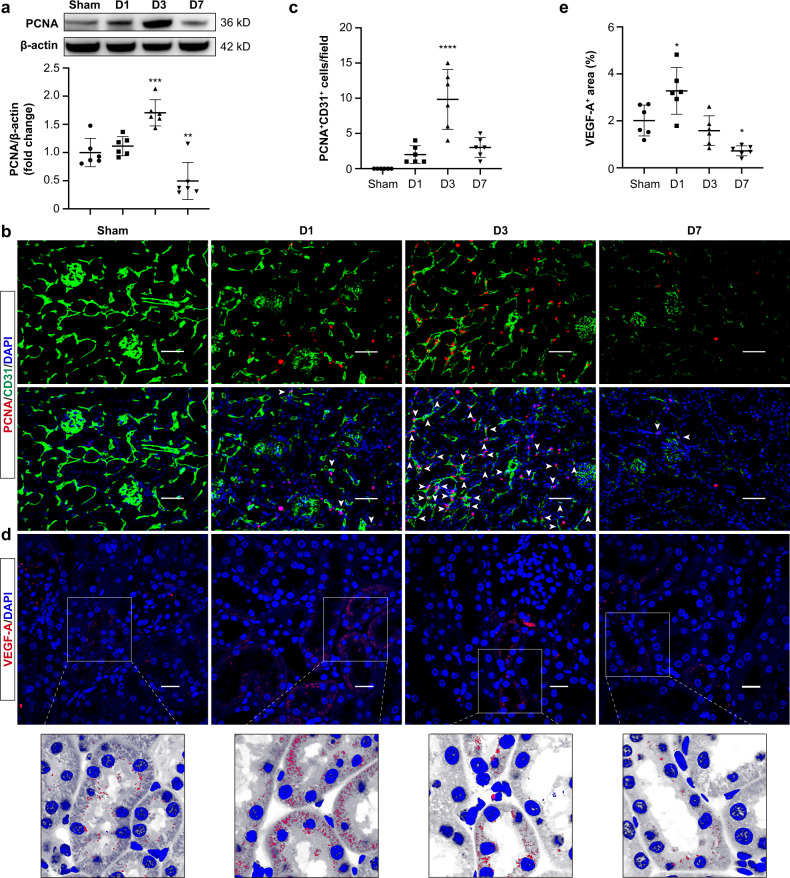
PTCs proliferation and tubular expression of VEGF-A are induced after I/R injury. **a** Western blotting analysis of PCNA in kidneys (*n* = 6). **b** Representative images of CD31- and PCNA-stained kidney sections. Scale bars, 100 μm (*n* = 6). **c** Quantification of PCNA^+^CD31^+^ cells (white arrowheads) on the basis of six mice, with at least 10 fields counted in each. **d** and **e** Representative confocal images of VEGF-A-stained kidney sections. Scale bars, 20 μm. Quantification on the basis of six mice, with at least ten sections counted in each. Zoom panels show the redistribution of VEGF-A from the cytoplasm to the basolateral side of TECs. Data are presented as means ± SD. *p < 0.05, **p < 0.01, ***p < 0.001, ****p < 0.0001 vs. Sham group. One-way ANOVA.

Endothelial proliferation that promotes angiogenesis is crucial for kidney regeneration after AKI. We first detected the peritubular capillary endothelial cell proliferation in the I/R kidney. Western blot analysis showed proliferating cell nuclear antigen (PCNA) protein was increased at day 1 and peaked at day 3 after I/R (Fig. 2a). Consistent with PCNA expression pattern, a considerable amount of proliferating endothelial cells was noted in the tubulointerstitium at day 3 by double labeling PCNA and CD31 (Fig. 2b and c). However, this still failed to rescue capillary rarefaction especially at day 7 in our experimental setting (Fig. 2b and c), indicating insufficient angiogenesis after AKI.

Because VEGF-A is the predominant pro-angiogenesis factor that supports the normal structure of PTCs^21^, we next analyzed its expression and distribution in kidneys by immunofluorescence staining. On day 1 after I/R injury, VEGF-A expression was significantly upregulated in tubules, and a remarkable redistribution of VEGF-A from the cytoplasm to the basolateral side of TECs was observed (Fig. 2d and e); however, the degree of tubular VEGF-A declined rapidly at day 3 and was even lower than baseline levels by day 7 (Fig. 2d and e). We hypothesized that the deficiency of tubular VEGF-A may cause insufficient angiogenesis and subsequent PTC rarefaction, and how tubular VEGF-A redistributed to the basolateral side acts on PTCs needs further clarification.

### I/R injury shifts tubular VEGF-A secretion from free form to sEV

**Fig. 3:**
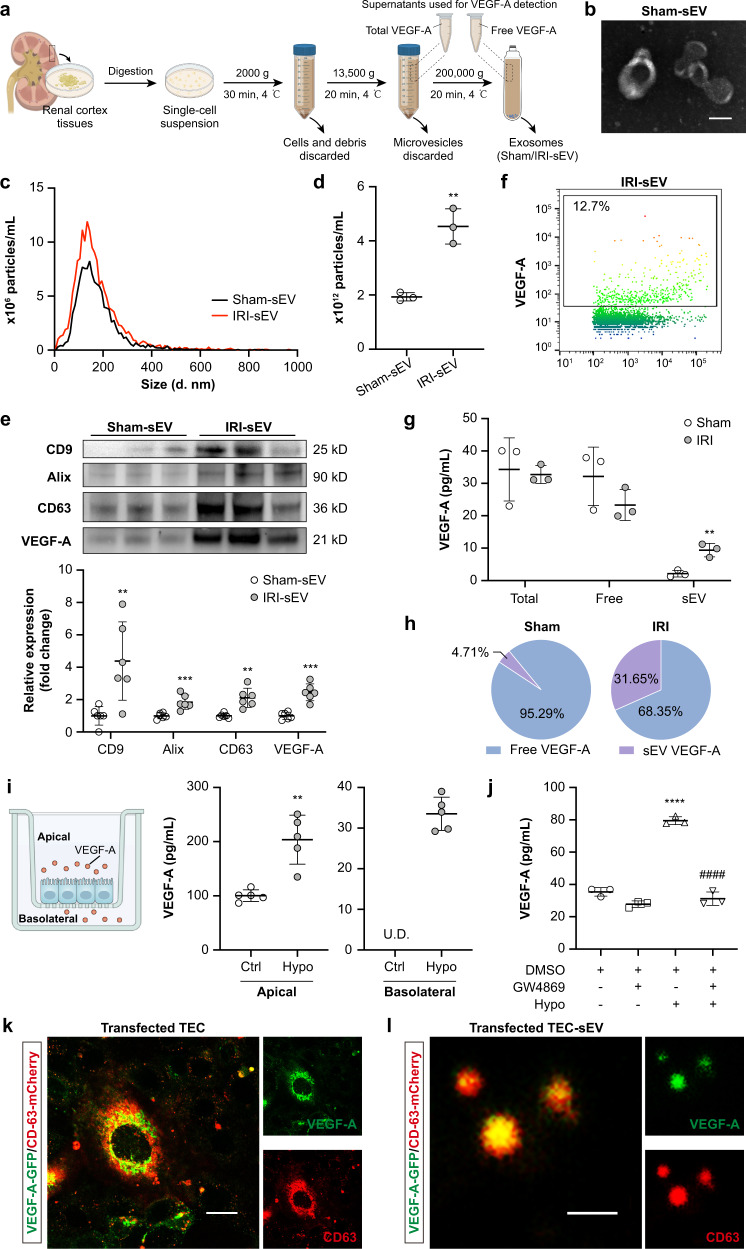
I/R injury shifts tubular VEGF-A secretion from free form to sEV. **a** Schematic illustration of the experimental design. In brief, renal sEV were purified using differential centrifugation from the same weight of digested renal cortex tissues. To quantify the VEGF-A levels in renal sEV, concentrations of total VEGF-A and free VEGF-A were first detected in the supernatants as indicated in the illustration, and then the content of VEGF-A in sEV was calculated by total VEGF-A subtracted from free VEGF-A. Schematic created with BioRender.com. **b** Representative TEM image of renal sEV purified from Sham mice. **c** Size distribution of sEV isolated from Sham (Sham-sEV) and IRI (IRI-sEV) mice. **d** Quantification of the particle concentrations of Sham-sEV and IRI-sEV (*n* = 3). **e** Western blotting analysis of sEV markers (CD9, Alix, and CD63) and VEGF-A in Sham-sEV and IRI-sEV (*n* = 6). **f** NanoFCM analysis of the VEGF-A + IRI-sEV. **g** ELISA analysis of the secreted VEGF-A in total, free, and sEV forms of the kidneys (*n* = 3). **h** Proportion of free VEGF-A and VEGF-A + sEV. **i** A transwell culture system was used to mimic the milieu of polarized TECs. mTECs were cultured in the upper chamber with or without hypoxia. The contents of VEGF-A in the upper (apical) and lower (basolateral) chambers were detected by ELISA (*n* = 5). **j** ELISA analysis of the VEGF-A showing hypoxia increased the secretion of VEGF-A in mTECs, which was inhibited by GW4869 administration (*n* = 3). mTECs were co-transfected with VEGF-A-GFP (green) and CD63-mCherry (red) to visualize the trafficking of VEGF-A + sEV under a confocal microscope. **k** Live-cell imaging showing the co-localization of VEGF-A and CD63. Scale bar, 20 μm. Related to Supplementary Movie 1. **l** Representative ultra-sensitive SIM image of the purified sEV from the transfected mTECs. Scale bar, 200 nm. Data are presented as means ± SD. **p < 0.01, ***p < 0.001, ****p < 0.0001 vs. Sham or Sham-sEV group (**a**–**h**), Ctrl (**i**), or DMSO^+^GW4869^-^Hypo^-^ group (**j**); ^####^*p* < 0.001 vs. DMSO^+^GW4869^-^Hypo^+^ group (**j**). Two-tailed Student’s *t* test (**d**–**i**), One-way ANOVA (**j**). U.D., undetected.

We explored the releasing pattern of tubular VEGF-A during AKI and focused on sEV, an important vector for secretory cytokines and growth factors^22^. In kidney tissues from biopsy of AKI patients, the co-localization of VEGF-A with CD63 was observed at the basolateral side of tubular cells, indicating that VEGF-A could be loaded into multivesicular bodies and released via vesicles (Supplementary Fig. 2). Then, we purified kidney sEV from the renal cortex from IRI-treated mice and tested VEGF-A as illustrated in Fig. 3a. The morphology of sEV was a typical membrane structure under a TEM (Fig. 3b). NTA showed that the mean diameters of sEV from sham (Sham-sEV) and IRI (IRI-sEV) kidneys were 171.4 nm and 161.3 nm, respectively (Fig. 3c). In addition, robust increasing amounts of IRI-sEV were detected than Sham-sEV using NTA (Fig. 3d) and Western blotting analysis of sEV markers (CD9, Alix, and CD63) (Fig. 3e). Interestingly, compared to Sham-sEV, increasing VEGF-A was detected in IRI-sEV purified from the same weight of renal tissues (Fig. 3e), which is consistent with previous report that VEGF can be packaged into extracellular vesicles (EVs)^23–25^. To further confirm VEGF-A expression in the single vesicle level, the sEV sample was analyzed by nano-flow cytometry, which identified 12.7% subpopulations of IRI-sEV expressing VEGF-A (Fig. 3f).

Subsequently, we compared the secreted VEGF-A that existed in sEV or in free form after I/R kidney injury by ELISA. Interestingly, although the total secreted VEGF-A from the supernatant depleted of microvesicles showed no significant difference between sham and AKI mice, we noted a trend of decrease in free VEGF-A and remarkably increased levels of sEV-VEGF-A at day 3 after injury (Fig. 3g). Correspondingly, the proportion of sEV-VEGF-A showed an approximately 7-fold increase in the ischemic kidney, accounting for 31.65% of the total VEGF-A (Fig. 3h). Moreover, treatment of sEV with proteinase K reduced the expression of VEGF-A, suggesting that VEGF-A is associated at the surface of EVs (Supplementary Fig. 3a). Therefore, I/R injury appeared to affect the secretion mechanism of VEGF-A, shifting it from the free form to sEV.

We further investigated the distribution and secretion of VEGF-A in TECs in vitro. A transwell culture system was employed, and VEGF-A in the supernatants from the upper and lower chamber that mimic the apical and basolateral side of tubules was tested by ELISA. Impressively, VEGF-A was undetectable in the lower chamber (basolateral) under normoxia, while hypoxia could strongly trigger VEGF-A secretion (Fig. 3i). To confirm whether hypoxia-induced VEGF-A was mainly secreted via sEV, we treated hypoxic TECs with GW4869 (20 μM), a potent neutral sphingomyelinases inhibitor known to inhibit sEV release^26^. As expected, GW4869 markedly reversed the up-regulation of VEGF-A in the medium of hypoxic TECs (Fig. 3j). Moreover, we transfected TECs with GFP-labeled VEGF-A and mCherry-labeled CD63 to visualize the intracellular trafficking and secretion of sEV-VEGF-A using confocal live-cell imaging. Interestingly, VEGF-A co-localized with CD63, trafficking together in the cytoplasm and likely releasing into the extracellular region (Fig. 3k and Supplementary Movie 1). Ultra-sensitive SIM confirmed the co-localization of the two proteins in hypoxia-sEV (Fig. 3l). These results demonstrated that hypoxia stress could efficiently augment the tubular secretion of sEV enriched with VEGF-A, and sEV-VEGF-A may be responsible for the increased peritubular capillary endothelial cell proliferation during AKI other than the free VEGF-A.

### Hypoxic TEC-derived sEV facilitate endothelial cell proliferation via VEGF-A signaling in vitro

**Fig. 4:**
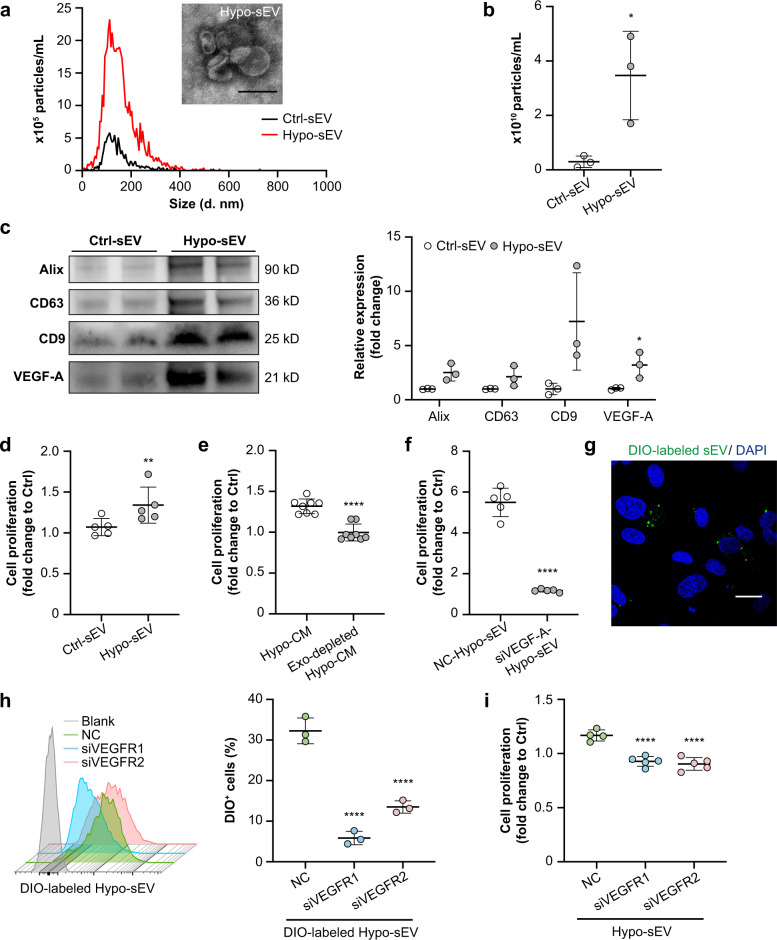
Hypoxic TEC-derived sEV facilitate endothelial cell proliferation via VEGF-A signaling in vitro. **a** Size distribution of sEV derived from HK-2 under normoxia (Ctrl-sEV) or hypoxia (Hypo-sEV). Representative TEM image of Hypo-sEV. **b** Quantification of the particle concentrations of Ctrl-sEV and Hypo-sEV (*n* = 3). **c** Western blotting analysis of sEV markers (Alix, CD63, and CD9) and VEGF-A in Ctrl-sEV and Hypo-sEV (*n* = 3). **d** Effects of purified Ctrl-sEV or Hypo-sEV (15 μg/ml) on the proliferation of HUVECs (*n* = 3). **e** Effects of the culture medium from hypoxic HK-2 cells with or without Hypo-sEV on the proliferation of HUVECs (*n* = 8). **f** HK-2 cells were transfected with siVEGF-A and were then exposed to hypoxia for 24 h. The conditioned Hypo-sEV with VEGF-A knockdown were purified for the analysis of their proliferative effects on HUVECs (*n* = 5). **g** A transwell culture system was employed to observe the sEV-mediated communication between HK-2 cells (upper chamber, DIO-stained) and HUVECs (lower chamber). Representative confocal image showing the uptake of DIO-labeled vesicles by HUVECs. Scale bar, 20 μm. **h** Flow cytometry analysis of the internalization of DIO-labeled Hypo-sEV by HUVECs with or without VEGFR1/VEGFR2 downregulation (*n* = 3). **i** Effects of VEGFR1/VEGFR2 knockdown of the recipient cells on the Hypo-sEV-mediated proliferation of HUVECs (*n* = 4 or 5). Data are presented as means ± SD. *p < 0.05, **p < 0.01, ****p < 0.0001 vs. Ctrl-sEV (**b**–**d**), Hypo-CM (**e**), NC-Hypo-sEV (**f**) or NC (**h**, **i**); Two-tailed Student’s *t* test (**b**–**f**), One-way ANOVA (**h**, **i**).

To explore the effects of tubular sEV on endothelial cells in vitro, we treated HUVECs with sEV from HK-2 cells with or without hypoxia (Ctrl-sEV or Hypo-sEV). Consistent with our in vivo findings, hypoxic HK-2 cells released much more sEV than normoxic cells, as evidenced by particle concentrations (Fig. 4a and b) and levels of sEV markers (Fig. 4c). Besides, increased expression of VEGF-A in hypoxia-sEV was verified as well (Fig. 4c). Importantly, Hypo-sEV showed a superior capacity to promote HUVECs proliferation than Ctrl-sEV (Fig. 4d), as well as the expression of inflammatory factors, including TNF-α, MCP-1, VCAM-1 and ICAM-1 (Supplementary Fig. 4). When we depleted sEV from the hypoxic culture medium (Hypo-CM), the proliferative effect of Hypo-CM was completely abrogated (Fig. 4e), suggesting that Hypo-sEV, rather than free VEGF-A or other soluble factors in the Hypo-CM, plays a critical role in HUVECs proliferation. In addition, Hypo-sEV derived from VEGF-A knockdown HK-2 cells (Fig. 4f) and loss of surface VEGF-A on Hypo-sEV with proteinase K treatment (Supplementary Fig. 3b) also reversed the proliferative effect on HUVECs significantly. These data demonstrated that Hypo-sEV containing VEGF-A derived from TECs contributes to endothelial proliferation.

To further investigate sEV-mediated cross-talk between hypoxic TECs and PTCs, HUVECs were seeded in the lower chamber of a transwell and co-cultured with DIO-stained HK-2 cells in the upper chamber. DIO-positive sEV released from labeled TECs could be internalized by endothelial cells (Fig. 4g). Flow cytometry showed that the uptake of Hypo-sEV significantly decreased in VEGFR1 or VEGFR2 down-regulated HUVECs, especially in the VEGFR1 knockdown group (Fig. 4h), indicating that VEGFR could affect the uptake of sEV by endothelial cells. Moreover, VEGFR1 or VEGFR2 knockdown markedly blocked Hypo-sEV-stimulated HUVEC proliferation (Fig. 4i). These results suggested that hypoxic TEC-derived sEV promoted the proliferation of endothelial cells via VEGF-A signaling.

### Knockdown of renal Rab27a reduces PTCs proliferation after ischemic injury in vivo

**Fig. 5:**
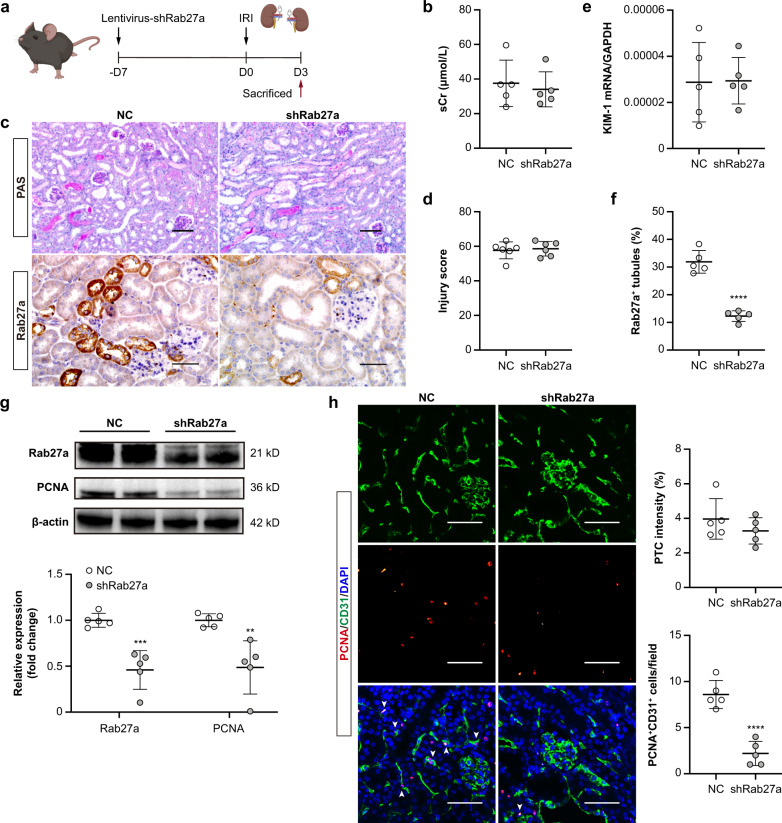
Knockdown of Rab27a reduces PTCs proliferation after ischemic injury. **a** Schematic diagram of the experimental design. Briefly, mice were subjected to a 28-min bilateral IRI at day 7-post lentiviral shRab27a injection, and were euthanized at 3 days after reperfusion. Schematic created with BioRender.com. **b** Effects of Rab27a knockdown on the serum creatinine after IRI (*n* = 5). **c** Representative images of PAS-stained kidneys (top) or Rab27a-immunostained kidney sections (bottom). Scale bars, 50 μm. **d** Quantification of kidney injury based on PAS staining (*n* = 5). **e** RT-qPCR analysis of KIM-1 mRNA levels in kidneys (*n* = 5). **f** Quantification of Rab27a^+^ tubules based on five mice, with at least ten sections counted in each. **g** Western blotting analysis of PCNA and Rab27a expression in kidneys (*n* = 5). **h** Representative confocal images of CD31- and PCNA-stained kidney sections. Scale bars, 50 μm. Quantification of PTC intensity and CD31^+^PCNA^+^ cells (white arrowheads) based on five mice, with at least ten sections counted in each. Data are presented as means ± SD. **p < 0.01, ***p < 0.001, ****p < 0.0001 vs. NC group. shRab27a, lentivirus containing shRNA against Rab27a. NC, negative control lentivirus. Two-tailed Student’s *t* test.

Rab27a is a member of the Rab family of small GTPases, which plays a well-established role in sEV release^27^. To confirm the effects of sEV on PTCs during ischemic injury in vivo, sEV secretion was inhibited by administration of lentiviral short hairpin RNA against Rab27a (shRab27a) (Fig. 5a). We found that Rab27a knockdown did not affect serum creatinine and kidney histological injury at day 3 post-I/R (Fig. 5b–f). However, a remarkable decrease of renal PCNA expression was noticed in shRab27a mice compared to NC group (Fig. 5g). Double-labeling with PCNA and CD31 revealed that endothelial proliferation of PTCs was suppressed in shRab27a mice at 3 days after I/R, and also a decreasing trend of PTCs density was noted (Fig. 5f). Thus, inhibition of sEV secretion by knockdown of Rab27a repressed proliferation of PTCs after AKI.

### VEGF-A + sEV treatment attenuates AKI-CKD transition in mice

**Fig. 6:**
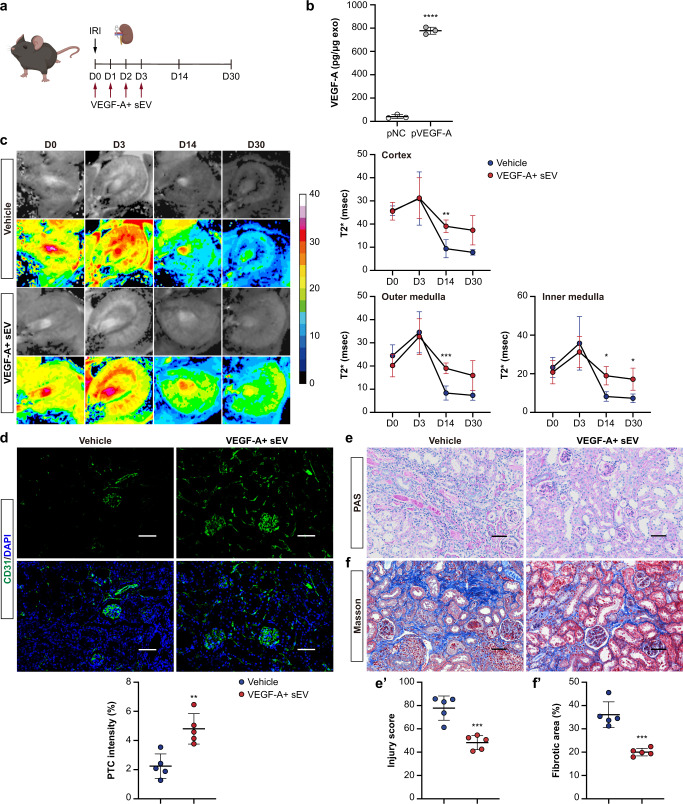
VEGF-A + sEV treatment attenuates AKI-CKD transition in mice. **a** Schematic diagram of the experimental design. Briefly, mice were subjected to 35 min of unilateral renal IRI, and VEGF-A + sEV (200 μg) or vehicle was administered upon reperfusion and continued every 12 h for seven times. Mice were euthanized at day 30-post IRI. Schematic created with BioRender.com. **b** ELISA analysis of VEGF-A concentration in sEV (*n* = 3). **c** Representative images of BOLD-MRI analysis of renal perfusion. Colored scale represents T2* relaxation time (in milliseconds). Quantification of T2* in renal cortex, outer medulla, and inner medulla (*n* = 5). **d** Representative confocal images of CD31-stained kidney sections. Quantification on the basis of five mice, with at least ten sections counted in each. **e** and **e’** Representative images of PAS staining and quantification of kidney injury. Scale bars, 50 μm (*n* = 5). **f** and **f’** Representative images of Masson trichrome staining and quantification of fibrotic area. Scale bars, 50 μm (*n* = 5). Data are presented as means ± SD. *p < 0.05, **p < 0.01, ***p < 0.001, ****p < 0.0001 vs. pNC (**b**) or Vehicle group (**c**, **d**, **e’**, **f’**). Two-tailed Student’s *t* test.

**Fig. 7:**
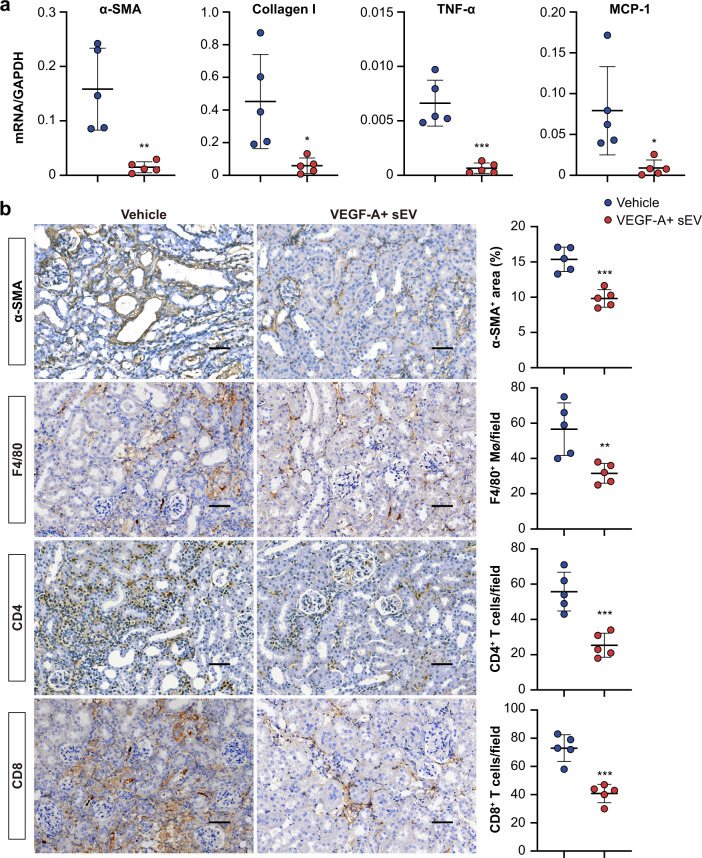
VEGF-A + sEV protects against I/R-induced chronic tubulointerstitial inflammation and fibrosis. **a** RT-qPCR analysis of α-SMA, Collagen I, TNF-α, and MCP-1 mRNA levels in kidneys at day 30-post I/R injury (*n* = 5). **b** Immunohistochemical analysis of α-SMA accumulation and infiltration of macrophages (F4/80) and T cells (CD4 and CD8) in kidney sections. Scale bars, 50 μm. Quantification on the basis of five mice with at least ten sections counted in each. Data are presented as means ± SD. *p < 0.05, **p < 0.01, ***p < 0.001 vs. Vehicle group. Two-tailed Student’s *t* test.

We found that, although renal tubules can secrete sEV-VEGF-A to promote peritubular endothelial cell proliferation after injury, the endogenous yield may be insufficient to support the full recovery of PTCs. Thus, we investigated the therapeutic potential of sEV-VEGF-A supplement on renal recovery following AKI. We transfected mTECs with a plasmid coding murine VEGF-A to generate sEV expressing high levels of VEGF-A (VEGF-A + sEV) (Supplementary Fig. 5). To permit long-term observations, a 35-min unilateral renal I/R injury was adopted, and VEGF-A + sEV was administered intravenously after reperfusion and continued every 12 h for seven times (Fig. 6a). ELISA assay confirmed that abundant VEGF-A protein was loaded into sEV (Fig. 6b). Then, we utilized a noninvasive technique, blood oxygen level-dependent (BOLD) imaging^28^, to monitor renal oxygenation after VEGF-A + sEV administration. BOLD-MRI showed that VEGF-A + sEV enhanced the T2* values of renal cortex, outer medulla, and inner medulla of the ischemic kidney, especially at days 14 and 30-post injury, suggesting improved renal perfusion after VEGF-A + sEV treatment (Fig. 6c). CD31 staining revealed that the loss of PTCs was markedly prevented by VEGF-A + sEV (Fig. 6d). Consistent with the improved microvasculature and renal perfusion, I/R injury-induced chronic tubulointerstitial damage and fibrosis, including tubular atrophy, cast formation, inflammatory cell infiltration, and extracellular matrix deposition, were significantly ameliorated by the treatment of VEGF-A + sEV (Fig. 6e and f). In parallel, mRNA expressions of α-SMA, Collagen I, TNF-α, and MCP-1 (Fig. 7a) and α-SMA accumulation, as well as the infiltration of macrophages and T cells (Fig. 7b), were also decreased in the kidneys of VEGF-A + sEV-treated mice. These findings suggested that early treatment with VEGF-A + sEV can prevent AKI to CKD progression via promoting PTCs repair.

## Discussion

**Fig. 8:**
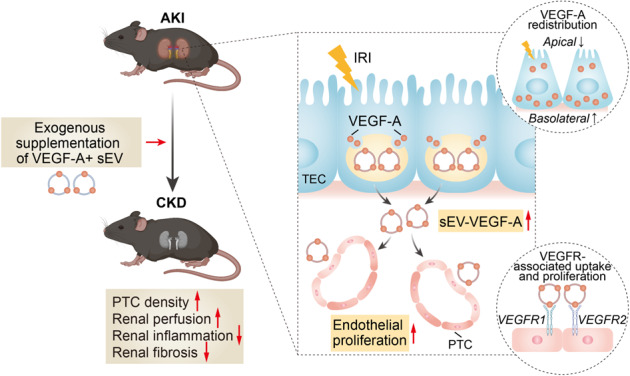
Working models propose tubular sEV-VEGF-A mediated tubule-PTCs communication, which functions as an intrinsic repair mechanism in ischemic AKI. We demonstrated that hypoxia triggers a shift of tubular VEGF-A secretion from the free form to sEV, favoring the endothelial proliferation of PTCs after ischemic AKI. Furthermore, supplementary treatment with VEGF-A + sEV efficiently ameliorated PTC rarefaction and protected against the chronic progression of AKI. Schematic created with BioRender.com.

Renal tubules and PTC network are closely related and interactive, working together to maintain normal kidney function. Disruption of these two structures can lead to the development and progression of various kidney diseases^29^. However, how they shape one another and affect kidney injury and repair has not been well elucidated. In the present study, we demonstrated that hypoxia triggers a shift of tubular VEGF-A secretion from the free form to sEV, favoring the endothelial proliferation of PTCs after ischemic AKI. Furthermore, supplementary treatment with VEGF-A + sEV efficiently ameliorated PTC rarefaction and protected against the chronic progression of AKI (Fig. 8).

Angiogenesis-related molecules derived from renal parenchymal cells are critical for the structure and function of renal capillary networks. Among them, VEGF-A produced by podocytes and TECs is an important pro-angiogenic factor in maintaining glomerular endothelial health^30^ and peritubular microvasculature integrity^28^, respectively in physiological conditions. Interestingly, localized regions of enhanced VEGF expression were noted in tubular cells but not glomerular podocytes as a compensatory mechanism to hypoxia, especially at the early stages of injury^31,32^. In this study, we found that tubular VEGF-A expression is significantly increased at the early stage of renal I/R injury, and an apparent redistribution of cytosolic VEGF-A to the basolateral aspect of TECs was also noted, which is consistent with the previous report by Kanellis et al.^33^. However, the fate of the redistributed VEGF-A and their secretion mode are poorly understood. It is known that some VEGF isoforms contain a signal peptide that allows for the translocation and export of the protein through the conventional secretion pathway^34–36^. Nevertheless, our study uncovered an unconventional protein secretion approach of tubular VEGF-A in response to I/R injury that renal tubules augment the release of VEGF-A via sEV other than in the free form during AKI. Indeed, it has been recognized that unconventional secretion related with vesicles may be induced upon cell stress for many proteins^37^. The sEV format of VEGF-A may benefit local accumulation compared to diffusible free ones and promoted angiogenesis effects on PTCs. In addition, VEGF in cancer cell-derived EVs have also been identified^23,26,38^, and tetraspanin CD63 might participate in the packaging of VEGF into cancer EVs^38^. However, the underlying mechanism of the specific sorting of tubular VEGF-A into sEV after AKI remains to be clarified.

Aside from the secreted proteins, sEV have emerged as an important signalosome for intercellular communication in diverse (patho-)physiological processes. Previous studies in both AKI and CKD experimental models have shown that sEV-mediated TEC-macrophage^17,18,39^ or TEC-fibroblast^40,41^ crosstalk can cause the progression of tubulointerstitial inflammation and fibrosis. Here, we demonstrated that TEC-derived sEV containing VEGF-A could be took up by PTCs, resulting in the proliferation of peritubular endothelial cells, which is beneficial to restore renal microvasculature. These findings suggest that tubular sEV are multifaceted, presenting different effects depending on recipient cells. Traditionally, VEGF-A secreted from TECs binds to and activates VEGFR2 to promote endothelial cell proliferation of PTCs^42,43^. Our study implied that both VEGFR1 and VEGFR2 are potential mediators that regulate the uptake and proliferative activity of sEV-VEGF-A on endothelial cells, indicating a new endothelial protection mechanism in sEV-VEGF-A-mediated epithelial-endothelial crosstalk.

Regenerative angiogenesis to maintain the vessel networks represents a promising treatment of kidney disease with hypoxia. VEGF-A is the most common therapeutic candidate and has been shown to improve PTC rarefaction and renal fibrosis in animal models of CKD^44,45^. Recently, the positive outcome of VEGF-A mRNA on skin blood flow in patients with type 2 diabetes further strengthens the potential of VEGF-A in therapeutic angiogenesis^46^. In the present study, inspired by the endogenous repair response of tubule-derived VEGF-A + sEV, we developed sEV overexpressing recombinant VEGF-A by engineering TECs and demonstrated a proof of concept: hijacking exogenous sEV as a nanocarrier to deliver VEGF-A to the kidney, which efficiently restored PTC density, reduced renal inflammation and fibrosis, and ultimately blocked AKI-to-CKD transition. Indeed, EVs have evolved as a focus technology in drug delivery and can be tailored to target specific renal cells^47–49^. Further refinement is still needed to enhance the PTC-targeting of VEGF-A + sEV to fully relieve the side-effect concerns.

In conclusion, we have demonstrated that sEV serve as a new secretion route of tubular VEGF-A, facilitating the endothelial proliferation of PTCs after I/R injury. Moreover, exogenous supplementation of VEGF-A + sEV was an effective therapeutic avenue to alleviate PTC rarefaction and halt the chronic progression of AKI. Our study provides a previously unrecognized mechanism of tubulovascular crosstalk via sEV-VEGF-A and will open new opportunities for VEGF-based therapeutic angiogenesis by exploiting sEV-mediated delivery.

## Methods

### Animal models

6–8-week-old Male C57BL/6 mice, purchased from Beijing Vital River Laboratory Animal Technology Co., Ltd., Beijing, China, were used for the experiments. All animal experiments were approved by the Institutional Animal Care and Use Committee of Southeast University (No. 20210302023). For the bilateral I/R injury, mice were subjected to ischemia by clamping the bilateral renal pedicles with non-traumatic microaneurysm clamps for 28 min followed by reperfusion of renal blood flow by removing the clamps as reported before^18^. Sham mice underwent the same surgical procedures, except that the renal pedicles were not clamped. Mice were sacrificed on days 1, 3, and 7 after surgery. All procedures were performed at a body temperature of 36.5–37.5 °C by a sensitive rectal probe. For the unilateral I/R injury, only the right renal pedicle was clamped for 35 min^48^. Exogenous VEGF-A + sEV (200 μg) or vehicle was administered intravenously after reperfusion and continued every 12 h for seven times. Mice were sacrificed on day 30 after I/R injury.

### Knockdown of Rab27a in vivo

To inhibit sEV secretion in the kidney, Rab27a was knocked down by the administration of 5 × 10^7^ TU of lentivirus carrying short hairpin RNA against Rab27a (shRab27a) (5′-GATGCACGCGTACTGTGAA-3′) or negative control (NC) (5′-TTCTCCGAACGTGTCACGT-3′) (GeneChem) via tail vein injection one week before I/R surgery.

### Blood oxygenation level-dependent magnetic resonance imaging (BOLD-MRI)

Mice were fasted for 6–8 h before BOLD-MRI and were then anesthetized through sevoflurane inhalation, and the respiration was maintained at about 30 breaths per minute during the entire examination. BOLD-MRI images were collected by using a multiecho T2*-weighted gradient-echo sequence with the following parameters: 100/4-52; 10 echo times with spacing time of 5.3 ms; 30° flip angle; section thickness, 1 mm; 256 * 256 matrix; FOV, 3.5 * 3.5 cm; four signals were acquired. The decay constants or T2* were calculated by fitting an exponential curve to individual voxels over the echo times. Regions of interest (ROIs) were posited manually on the cortex, the outer medulla, and the inner medulla of both kidneys on T2 maps. The relative T2* changes were obtained before and after renal I/R injury at days 3, 14, and 30 and were then calculated by a radiologist who was blinded to ROIs^28^.

### Kidney histology and quantification

Kidneys were fixed with 4% paraformaldehyde, embedded in paraffin, and sectioned to 4 μm thickness for Periodic Acid-Schiff (PAS), Masson’s trichrome, immunohistochemistry, or immunofluorescence staining. To evaluate the kidney injury score, ten random kidney sections from each mouse were assessed based on PAS staining and each image was randomly divided into 100 graticule grids. Histology of tubular in each grid was semiquantitative assessed as follows: 0 = normal histology; 1 = tubular cell swelling, exfoliation, or necrosis, brush border loss, cast formation, tubular dilation, tubular atrophy, immune cell infiltration^47^. The injury score was calculated by adding all 100 grids scores from each image and then taking the average of ten random images. For renal fibrosis analysis, at least five random sections per mouse were assessed based on Masson’s trichrome staining and the percentage of fibrotic area as defined by blue staining was calculated.

For immunostaining, fixed kidney sections were deparaffinized and rehydrated, and primary antibodies against α-SMA (1:200, ab-5694, Abcam), CD4 (ZM-0418, ZSGB-Bio), CD8 (ZA-0508, ZSGB-Bio), F4/80 (1:200, ab-6640, Abcam), CD63 (1:200, ab193349, Abcam), CD31 (1:200; ab182981, Abcam), VEGF-A (1:200, sc-7269, Santa Cruz Biotechnology), and PCNA (1:200, sc-56, Santa Cruz Biotechnology) were used for overnight incubation, followed by incubating with the corresponding secondary antibodies. Regarding to PTC intensity, the percentage of CD31^+^ area (green staining) was quantified using Image Pro Plus image analysis system.

In addition, renal biopsy was obtained from a group of five AKI patients approved by Ethical Committee of Zhong Da Hospital Southeast University. Patients were enrolled into AKI group according to KDIGO criteria^50,51^. Primary antibodies against VEGF-A (1:200, sc-7269, Santa Cruz Biotechnology) and CD63 (1:200, GB11620, Servicebio) were used for immunofluorescence staining.

### Cell culture and intervention

HK-2 cells were obtained from ATCC. Immortalized mouse TECs (mTECs) were a gift from J. B. Kopp, National Institutes of Health. Both HK-2 and mTECs were cultured in DMEM/F-12 (Hyclone) supplemented with 10% fetal bovine serum (ScienceCell) and 1% penicillin-streptomycin (Gibco). Primary human umbilical vein endothelial cells (HUVECs) were extracted from umbilical cord segments via Collagenase I digestion and cultured with endothelial cell medium (ECM) (ScienceCell) containing 5% fetal bovine serum and 1% penicillin‐streptomycin (ScienceCell). HUVECs at passages 3–8 were used. All cells were cultured in a humidified atmosphere of 95% air and 5% CO_2_ at 37 °C. For hypoxic treatment, cells were cultured in glucose- and serum-free medium and placed in a hypoxic chamber (Thermo Scientific) with 1% O_2_/5% CO_2_ for 24 h.

A transwell system was used to analyze sEVs-mediated tubular cell-endothelial cell communication. A total of 4 × 10^4^ HK-2 cells and HUVECs were seeded in the upper and lower chamber, respectively. HK-2 cells were stained with DIO dye (5 mg/ml) for 30 min at 37 °C, and the free dye was completely washed away with PBS three times. Next, the transwell system was placed in the hypoxic chamber of 1% O_2_/5% CO_2_ for 12 h. DIO-labeled vesicles internalized by HUVECs were observed using confocal microscopy and quantified by flow cytometry analysis.

To knock down VEGF-A and VEGF receptor (VEGFR) 1/2 in HK-2 cells and HUVECs, respectively, small interfering RNAs (siRNAs) targeting VEGF-A (5′-GCAGCUACUGCCAUCCAAUTT-3′, 5′-AUUGGAUGGCAGUAGCUGCTT-3′), VEGFR1 (5′-CGUGGCUACUCGUUAAUUATT-3′, 5′UAAUUAACGAGUAGCCACGTT-3′), VEGFR2 (5′-GCCACCAUGUUCUCUAAUATT-3′, 5′UAUUAGAGAACAUGGUGGCTT-3′), and scrambled siRNA as a negative control (NC) were synthesized by GenePharma Co., Ltd. The transfection was performed using Lipofectamine 2000 (Invitrogen) according to the manufacturer’s protocol.

### Live-cell imaging and ultra-sensitive structured illumination microscopy (SIM)

To visualize VEGF-A secretion in sEV, we concurrently transfected mTECs with plasmids encoding GFP-labeled VEGF-A or mCherry-labeled CD63 (Genechem) using Lipofectamine 3000 (Invitrogen) according to the manufacturer’s protocol. After 24-h transfection, cells were observed under an FV3000 confocal microscope for live-cell imaging. In addition, the labeled sEV were purified from the supernatants to further verified GFP-VEGF-A and mCherry-CD63 expression by an ultra-sensitive SIM (Hessian SIM, Nanjing Brain Observatory).

### sEV purification and characterization

sEV were isolated from the culture supernatants or kidney lysates by differential centrifugation. To extract sEV from the kidney, renal cortex tissues were digested with collagenase IV (17104-019, Gibco) and trypsin (25200-056, Gibco) for 120 min at 37 °C, followed by termination with sEVs -free serum, and were subsequently subjected to sequential centrifugation steps^17,39^. In brief, the collected samples were first centrifuged at 2000 *g* for 20 min and 13,500 *g* for 20 min to remove cells, debris, and large vesicles, respectively. The resulting supernatants were then centrifuged for 2 h with a Type 70 Ti rotor (Beckman Optimal-100 XP) at 200,000 *g*, 4 °C. The pellets were washed one time and resuspended in sterile PBS and stored at −80 °C for further analysis.

Purified sEV samples were pipetted on 200-mesh nickel grids, stained with 2% phosphotungstic acid for 5 min and air dried for the morphology analysis with a transmission electron microscopy (TEM). The size distribution of sEV was detected by nanoparticle tracking analysis (NTA) using the ZetaView PMX 110 (Particle Metrix).

To remove the surface proteins on sEVs, purified sEVs were incubated with 20 μg/ml of proteinase K (BioFroxx, 1124MG100) at 37 °C for 1 h^52^. Protease activity was stopped by the addition of 5 mM phenylmethylsulfonyl fluoride for 10 min at room temperature.

### NanoFCM analysis of sEV-VEGF-A

sEV isolated from the I/R kidney were incubated with the antibody against VEGF-A (1:200, sc-7269, Santa Cruz) in 100 μL PBS at 4 °C overnight. After washing with PBS, sEV were recovered at 100,000 *g* after 2 h of ultracentrifugation and were then incubated with the Alexa Fluor 555-conjugated secondary antibody (Abcam), followed by another ultracentrifugation to pellet sEV. Before measurements, the standard sample was used for parameter calibration. The data were collected by nano-flow cytometry (Flow NanoAnalyzer). The positive and negative gating on VEGF-A was automatically distinguished by NanoFCM.

### Preparation of exogenous VEGF-A + sEV

To overexpress VEGF-A in mTEC, transfection was performed using Lipofectamine 3000 (Invitrogen) with plasmids encoding VEGF-A-GFP (Genechem) according to the manufacturer’s protocol. After 8 hours of transfection, the medium was replaced with fresh DMEM/F-12 for another 40-hour culture, and then the DMEM/F-12 medium was collected and subjected to differential ultracentrifugation. The resulting sEV pellets were washed one time with PBS, followed by filtering with a 0.22-μm filter, and finally were harvested in PBS for the following experiments.

### ELISA analysis of VEGF-A

VEGF-A was determined by the mouse VEGF-A ELISA Kit (DY008, R&D Systems) according to the manufacturer’s instructions. To examine VEGF-A secretion models, kidney was digested and processed via differential centrifugation to obtain the total VEGF-A and free VEGF-A fraction as depicted in Fig. 3A. The content of VEGF-A in sEV was calculated by total VEGF-A subtracted from free VEGF-A. For detection of purified sEV carried with VEGF-A, samples were lysed in radioimmunoprecipitation assay buffer before ELISA analysis.

### Western blotting

Western blotting analysis was conducted as reported before^39^. Rabbit anti-CD9 (1:1000, ab92726, Abcam), mouse anti-CD63 (1:1000, ab193349, Abcam), mouse anti-Rab27a (1:1000, ab55667, Abcam), rabbit anti-CD31 (1:1000, ab28364, Abcam), mouse anti-Alix (1:1000, sc-53540, Santa Cruz Biotechnology), anti-PCNA (1:1000, sc-56, Santa Cruz Biotechnology), anti-VEGF-A (1:1000, sc-7296, Santa Cruz Biotechnology), mouse anti-β-actin(1:4000, sc-47778, Santa Cruz Biotechnology) antibodies were used. We have included original western blot chemiluminescent images with corresponding light micrographs showing molecular weight markers for all western blots in Supplementary Fig. 7.

### HUVECs proliferation assay

To assess the effect of tubule epithelial cells derived sEV on HUVECs proliferation, the same species of cell line, HK-2 cells were used in this part. HK-2 cells were grown to 70–80% confluence and cultured in serum-free medium for 24 h. Then HK-2 cells were stimulated with hypoxia or normoxia as a control. sEV released from HK-2 cells were purified and washed completely in 20 ml of PBS, collected by ultracentrifugation as described above. sEV released from per 3 × 10^6^ HK-2 cells were added to HUVEC (5000 cells/well) for 12 h. Besides, sEV were depleted by ultracentrifugation from hypoxic culture medium (Hypo-CM) which was then applied to HUVEC to detect the proliferative effect of Hypo-CM with or without sEV. Cell Counting Kit-8 (CCK8) (CK04, Dojindo Laboratories) assay was used to analyze the proliferation of HUVECs. HUVECs were seeded in 96-well cell culture plates (5000 cells/well), followed by incubating with the CCK8 reagent at 37 °C for 2 h according to the manufacturer’s instructions. The absorbance at 450 nm was measured using a microplate reader (STNERGY/H4, BioTek).

### Quantitative real-time PCR

Total RNA was extracted from renal tissues or cells using TRIzol (Takara) and then reverse-transcribed using PrimeScript RT reagent kit (Takara) following the manufacturer’s instructions. Real-time quantitative PCR (RT-qPCR) was performed using TB Green Premix Ex Taq (Takara) on a 7300 PCR System (Applied Biosystems). The relative expression of mRNA was normalized to GAPDH levels. All the primers for RT-qPCR are listed in Table 1.

**Table 1:** Primers used in this study.

Gene	Forward (5′–3′)	Reverse (5′–3′)
Mouse TNF-α	CATCTTCTCAAAATTCGAGTGACAA	TGGGAGTAGACAAGGTACAACCC
Mouse CCL2	CTTCTGGGCCTGCTGTTCA	CCAGCCTACTCATTGGGATCA
Mouse ICAM-1	GTCTGCACCCAGTGCTAGTG	TGGATACCTGAGCATCACCA
Mouse VCAM-1	TGATTGGGAGAGACAAAGCA	AGCTCAACACAAGCGTGGAT
Mouse KIM-1	TCAGAAGAGCAGTCGGTACAAC	TGTAGCTGTGGGCCTTGTAGT
Mouse α-SMA	CAGCAAACAGGAATACGACGAA	AACCACGAGTAACAAATCAAAGC
Mouse Collagen-I	GTCAGACCTGTGTGTTCCCTACTCA	TCTCTCCAAACCAGACGTGCTTC
Mouse GAPDH	GCATGGCCTTCCGTGTTC	GATGTCATCATACTTGGCAGGTTT
Human VEGFR1	ATCATTCCGAAGCAAGGTGT	TTTCTTCCCACAGTCCCAAC
Human VEGFR2	GGAGCTTAAGAATGCATCCTTG	GATGCTTTCCCCAATACTTGTC
Human GAPDH	CTCTGCTCCTCCTGTTCGAC	GCGCCCAATACGACCAAATC
Human TNF-α	CCTCTCTCTAATCAGCCCTCTG	GAGGACCTGGGAGTAGATGAG
Human GAPDH	CTCTGCTCCTCCTGTTCGAC	GCGCCCAATACGACCAAATC
Human ICAM-1	CACAGTCACCTATGGCAACG	GCCGGAAAGCTGTAGATGGT
Human VCAM-1	ATACCCTCCCAGGCACACAC	GGTGCTGCAAGTCAATGAGA
Human CCL2	CCTTCATTCCCCAAGGGCTC	GGTTTGCTTGTCCAGGTGGT

### Statistical analysis

Data were presented as mean ± SD. Statistical analysis was carried out using *t* test or one-way analysis of variance (ANOVA) in Prism 5.0 GraphPad Software (San Diego, CA). *P* < 0.05 was considered statistically significant.

## Supplementary information

Supplemental Material

Supplementary Movie 1

## Data Availability

All data are available from the corresponding author upon reasonable request.
