# Supplementary material for: Tubular epithelial cells-derived small extracellular vesicle-VEGF-A promotes peritubular capillary repair in ischemic kidney injury
Source: NPJ Regen Med. 2022 Dec 17;7:73. doi: 10.1038/s41536-022-00268-x (PMC9759551; doi:10.1038/s41536-022-00268-x)
Supplement: Supplementary file 1 — Supplemental Material [file 41536_2022_268_MOESM1_ESM.pdf]

Supplementary materials

Tubular epithelial cells-derived small extracellular vesicle-VEGF-A promotes peritubular capillary repair in ischemic kidney injury

Xin Zhong[1†], Tao-Tao Tang[1†], An-Ran Shen[1], Jing-Yuan Cao[1], Jing Jing[1], Cui Wang[1], Xiao-Xiao Zhu[1], Yi Wen[1], Zuo-Lin Li[1], Bin Wang[1], Suo-Fu Qin[2], Bi-Cheng Liu[1]*, Lin-Li Lv[1]*

[1] Institute of Nephrology, Zhong Da Hospital, Southeast University School of Medicine; 87 Ding Jia Qiao Road, Nanjing, China.

[2]Kexing Biopharm Co., Ltd; Floor 15-19, Building B, Chuangyi Technology Building, No.198, Keji Middle 1st Road, Nanshan, Shenzhen, China.

†These authors contributed equally to this work

*Corresponding author: Lin-Li Lv (email: lvlinli@seu.edu.cn) or Bi-Cheng Liu (email: liubc64@163.com), Institute of Nephrology, Zhong Da Hospital, Southeast University School of Medicine; 87 Ding Jia Qiao Road, Gulou District, Nanjing, Jiangsu Province, China. Tel: 0086 25 83262422, Fax: 0086 25 83262422

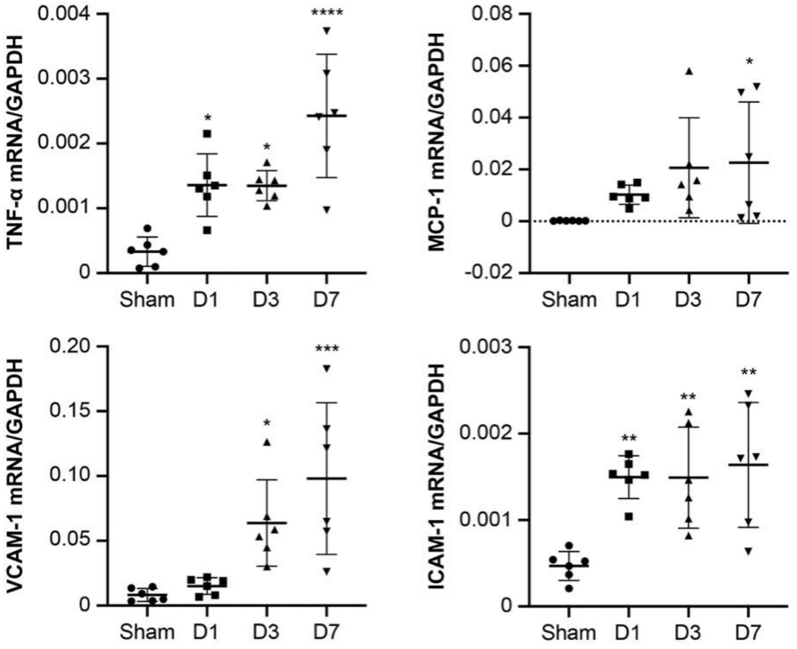

Supplementary Figure 1. RT-qPCR analysis of TNF-α, MCP-1, VCAM-1, and ICAM-1 mRNA levels in the kidneys(n=6). Data are presented as means ± SD. * p<0.05, ** p<0.01, *** p<0.001, **** p<0.0001 vs. Sham group. One-way ANOVA.

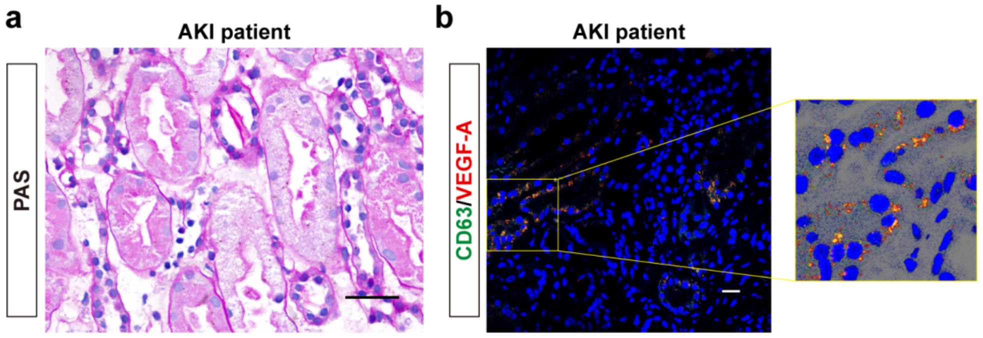

Supplementary Figure 2. Representative images of PAS-stained kidney sections (**a**) and VEGF-A and CD63-stained kidney sections (**b**) from biopsy of AKI patients. Scale bars, 100 μm (a); 20 μm (b).

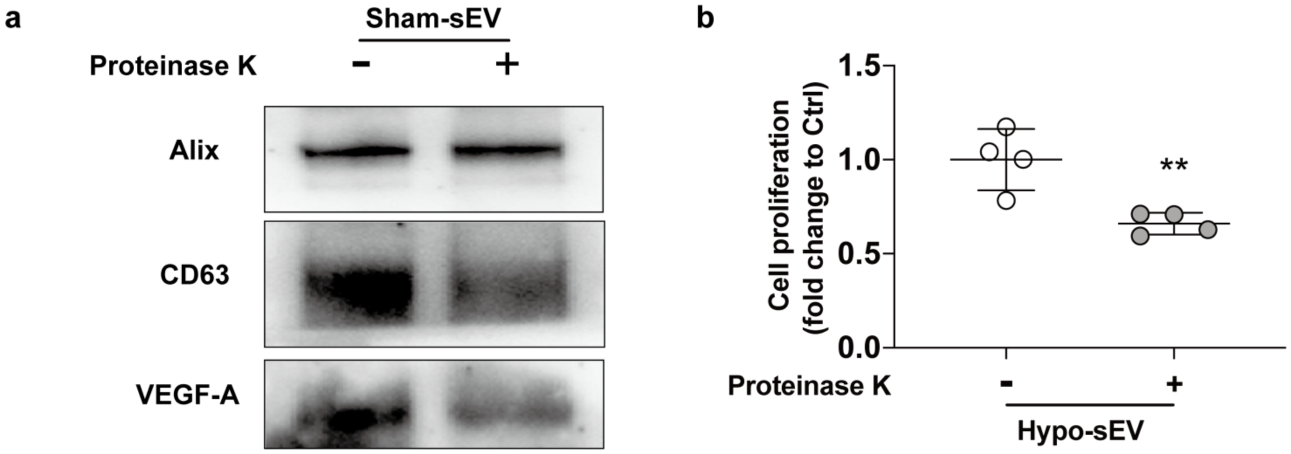

Supplementary Figure 3. (**a**) Western blotting analysis of Alix, CD63, and VEGF-A on Sham-sEV treated with or without proteinase K. (**b**) Effects of proteinase K-treated Hypo-sEV on the proliferation of HUVECs (n=4). Data are presented as means ± SD. ** $p<0.01$. Two-tailed Student's *t* test .

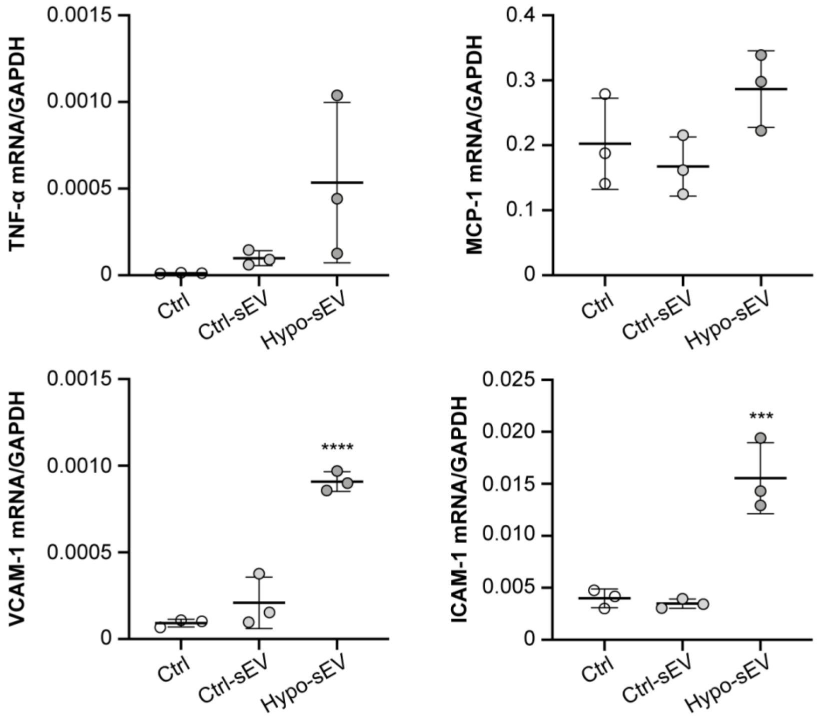

Supplementary Figure 4. RT-qPCR analysis of TNF-α, MCP-1, VCAM-1 and ICAM-1 mRNA expressions in HUVECs treated with Ctrl-sEV or Hypo-sEV (15 μg/ml) for 12 hours (n=3). Data are presented as means ± SD. *** $p<0.001$ vs. Ctrl group. Statistical One-way ANOVA.

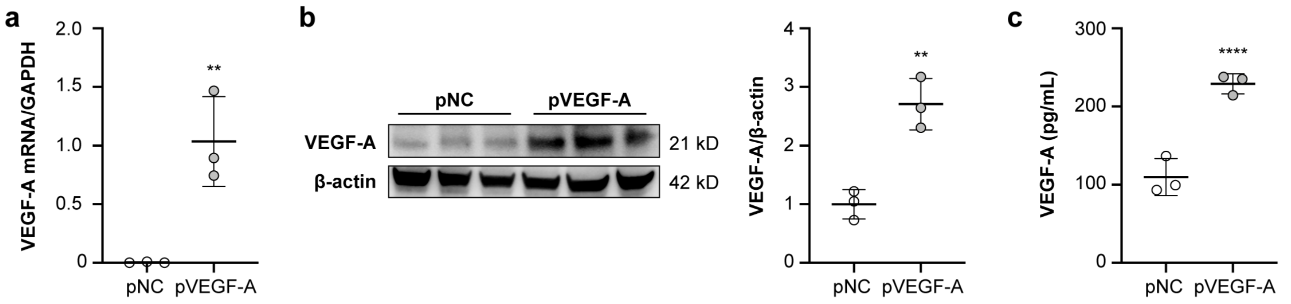

Supplementary Figure 5. mTECs were transfected with GFP-tagged VEGF-A to generate VEGF-A overexpressing sEVs. (**a**) RT-qPCR and (**b**) Western blotting analysis of VEGF-A expression in mTECs after transfection. (**c**) ELISA analysis of VEGF-A protein in the supernatants of transfected mTECs(n=3). Data are presented as means ± SD. ** p<0.01, **** p<0.0001 vs. pNC. Two-tailed Student's *t* test.

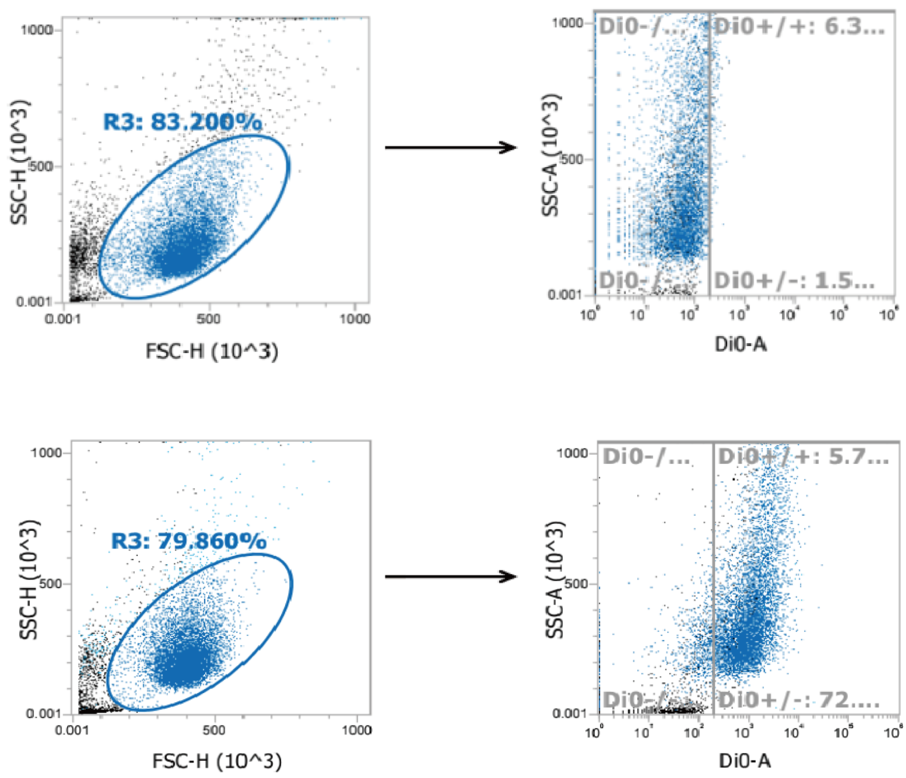

Supplementary Figure 6. FACS sequential gating strategy of Fig. 4H. Events were gated for single events followed by gating for DIO- or DIO+ HUVECs.

Unedited images for Fig. 2a

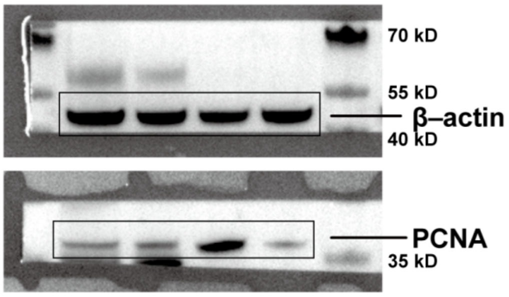

Unedited images for Fig. 3e

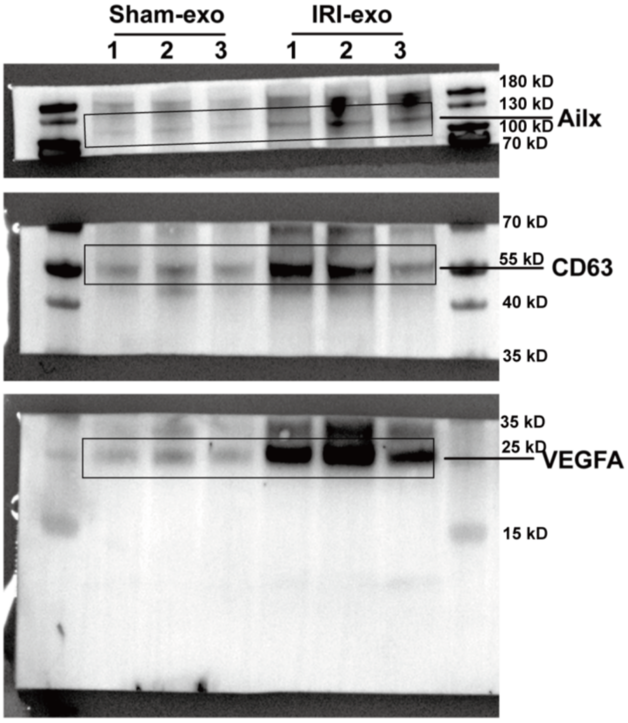

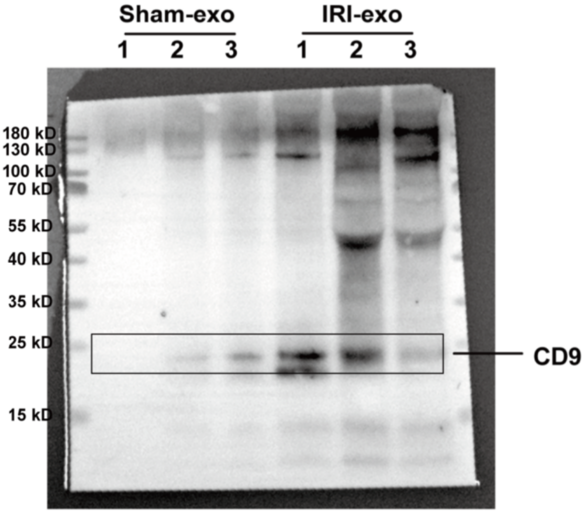

Unedited images for Fig. 4c

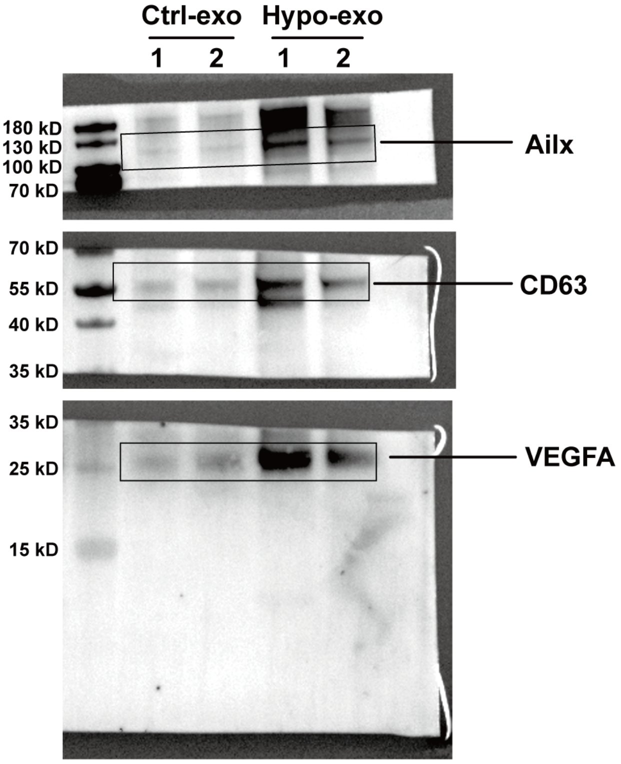

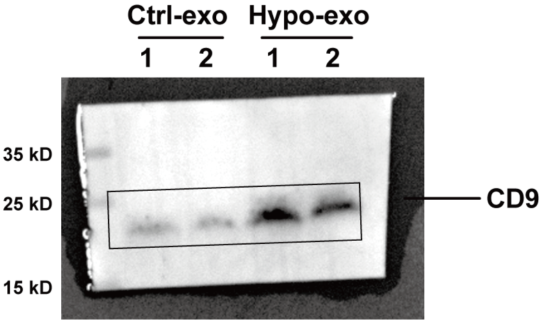

Unedited images for Fig. 5g

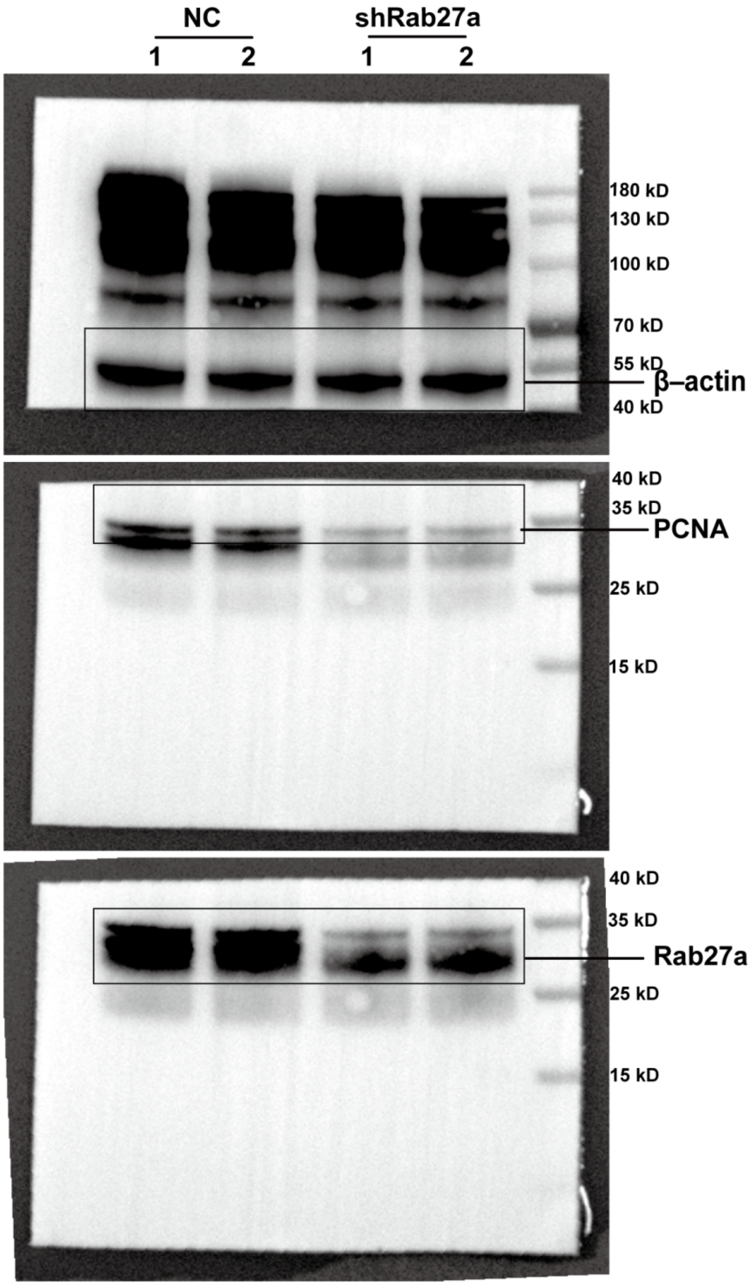

Unedited images for Supplementary Figure 5b

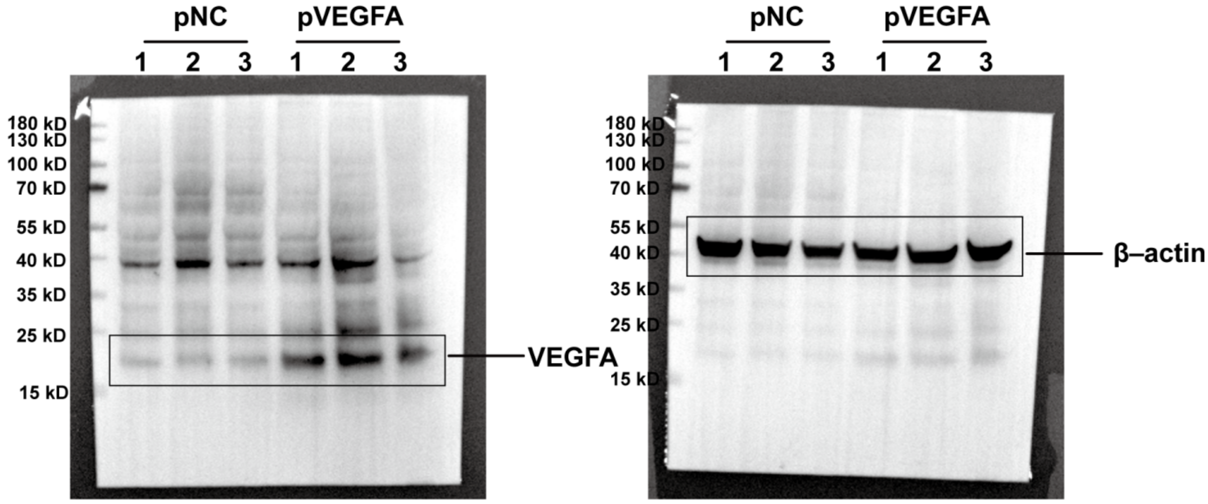

Supplementary Figure 7. The uncropped and unprocessed images of western blots shown in the manuscript.
